# Common spatiotemporal processing of visual features shapes object representation

**DOI:** 10.1038/s41598-019-43956-3

**Published:** 2019-05-20

**Authors:** Paolo Papale, Monica Betta, Giacomo Handjaras, Giulia Malfatti, Luca Cecchetti, Alessandra Rampinini, Pietro Pietrini, Emiliano Ricciardi, Luca Turella, Andrea Leo

**Affiliations:** 1 Momilab, IMT School for Advanced Studies Lucca, 55100 Lucca, Italy; 2 Center for Mind/Brain Sciences (CIMeC), University of Trento, 38068 Trento, Italy

**Keywords:** Computational neuroscience, Sensory processing, Visual system

## Abstract

Biological vision relies on representations of the physical world at different levels of complexity. Relevant features span from simple low-level properties, as contrast and spatial frequencies, to object-based attributes, as shape and category. However, how these features are integrated into coherent percepts is still debated. Moreover, these dimensions often share common biases: for instance, stimuli from the same category (e.g., tools) may have similar shapes. Here, using magnetoencephalography, we revealed the temporal dynamics of feature processing in human subjects attending to objects from six semantic categories. By employing Relative Weights Analysis, we mitigated collinearity between model-based descriptions of stimuli and showed that low-level properties (contrast and spatial frequencies), shape (medial-axis) and category are represented within the same spatial locations early in time: 100–150 ms after stimulus onset. This fast and overlapping processing may result from independent parallel computations, with categorical representation emerging later than the onset of low-level feature processing, yet before shape coding. Categorical information is represented both before and after shape, suggesting a role for this feature in the refinement of categorical matching.

## Introduction

**Figure 1:**
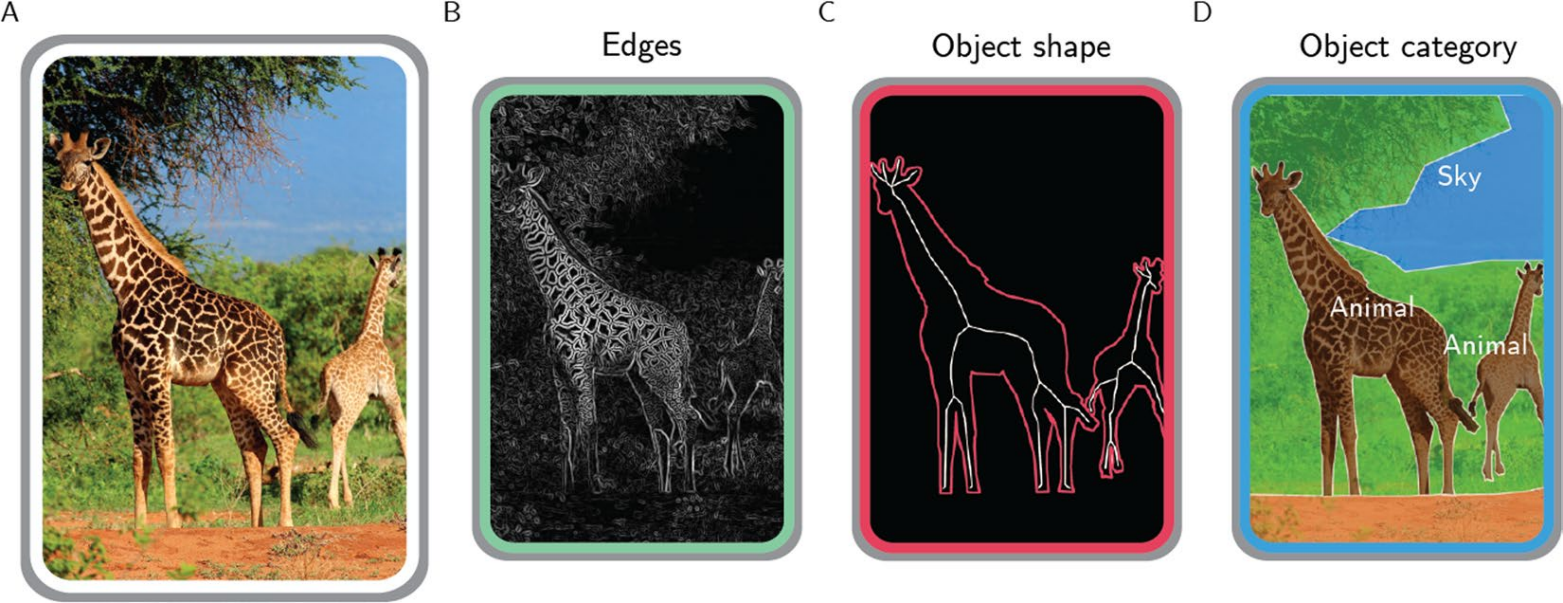
Different representations of a natural image. A real-world scene (**A**), depicting two giraffes in the savannah, can be defined by its edges (**B**), by the shape of the giraffes (**C**) and also by the categorical information it conveys (**D**). Photo taken from http://pixabay.com, released under Creative Commons CC0 license.

To make sense of the surrounding environment, our visual system relies on different transformations of the retinal input^1^. Just consider Fig. 1A. As any natural scene, this image is defined by a specific content of edges and lines. However, biological vision evolved to disclose the layout of discrete objects, hence the two giraffes in the foreground emerge as salient against the background, and the distinct contents pertaining to edges, shape, texture, and category contribute together to object perception.

Actually, each feature of Fig. 1B–D is processed across the whole visual system. The primary visual cortex (V1) provides an optimal encoding of natural image statistics based on local contrast, orientation and spatial frequencies^2,3^, and these low-level features significantly correlate with brain activity in higher-level visual areas^4,5^. Nonetheless, occipital, temporal and parietal modules also process object shape^6–9^ and categorical knowledge^10–12^.

Although all these features are relevant to our brain, their relative contribution in producing discrete and coherent percepts has not yet been clarified. In general, these different dimensions are interrelated and share common biases (i.e., are collinear), thus limiting the capability to disentangle their specific role^13^. For instance, categorical discriminations can be driven either by object shape (e.g., tools have peculiar outlines) or spatial frequencies (e.g., faces and places have specific spectral signatures^14^:). Consequently, object shape and category are processed by the same regions across the visual cortex, even when using a balanced set of stimuli^15^. Even so, the combination of multiple feature-based models describes brain object representations better than the same models tested in isolation. For instance, a magnetoencephalography (MEG) study found that combining low-level and semantic features improves the prediction accuracy of brain responses to viewed objects, suggesting that semantic information integrates with visual features during the temporal unfolding of object representations^16^.

To investigate the spatiotemporal dynamics of object processing, we combined model-based descriptions of pictures, MEG brain activity patterns and a statistical procedure (Relative Weights Analysis; RWA^17^,) that mitigate the effects of common biases across different dimensions. We ultimately determine the relative contribution across space and time of multiple feature-based representations – i.e., low-level, shape and categorical features - in producing the structure of what we perceive. First, a low-level description of the stimuli was grounded on features extracted by the early visual cortex (i.e., image contrast and spatial frequencies). Second, since shape is critical to interact with the surrounding environment^18^, we relied on a well-assessed, physiologically-motivated description of shape, i.e., the medial axis^19^. Finally, objects were also distinctively represented according to their superordinate categories.

To anticipate, we observed fast (100–150 ms) and overlapping representations of low-level properties (contrast and spatial frequencies), shape (medial-axis) and category in posterior sensors. These results may be interpreted as macroscale dynamics resulting in independent parallel processing, and may also suggest a role for shape in the refinement of categorical matching.

## Results

**Figure 2:**
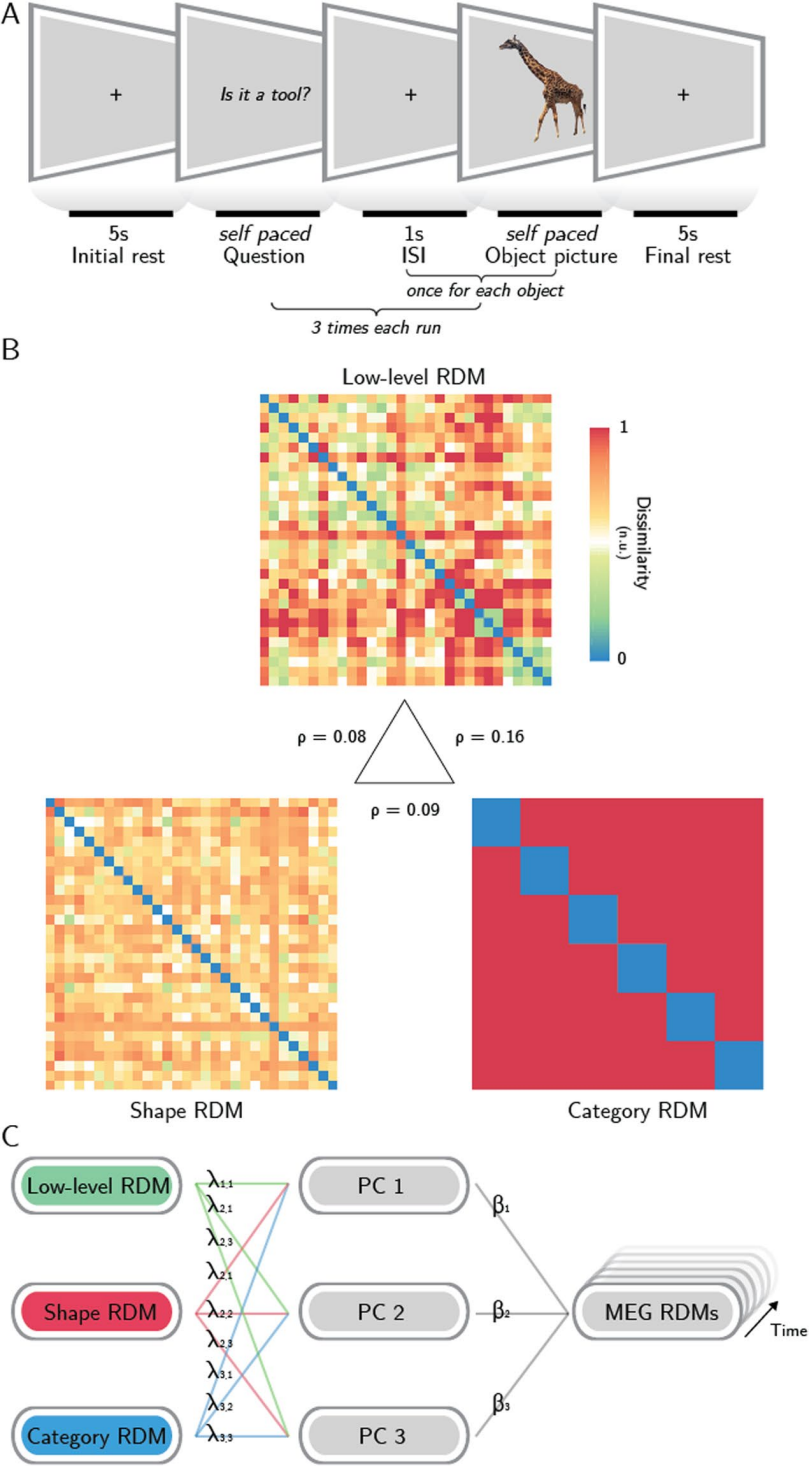
Methodological pipeline. (**A**) Experimental design: subjects were asked to attend thirty object pictures during a semantic judgment task. (**B**) representational dissimilarity matrices (RDMs) of three models (low-level features, shape and category) were employed to predict the MEG representational geometry – in the central triangle, Spearman correlation values between models are reported. With Relative Weights Analysis (**C**), MEG RDMs were predicted using three orthogonal principal components (PCs 1–3) obtained from the models, and the resulting regression weights were back-transformed to determine the relative impact of each model on the overall prediction when controlling for the impact of model collinearity (see Methods). Photo taken and edited from http://pixabay.com, released under Creative Commons CC0 license.

We employed the Relative Weights Analysis^16^ to reveal the proportional contribution of low-level, shape and category feature models in predicting time resolved representational geometries derived from MEG data, recorded from subjects attending to pictures representing thirty different stimuli from six semantic categories (Fig. 2).

The possible transformations of retinal input were described at three canonical steps of the object processing hierarchy, grounded on previous neurophysiological investigations. A first low-level model was computed by filtering the stimuli with a bank of Gabor filters: this model captures the arrangement of spatial frequencies in a V1-like fashion^2^. Then, as in previous neuroimaging investigations on the same topic^9,20^, we described object shape as its medial-axis transform^19^, that roughly describes an object as its skeleton, with each object part captured by a different branch. And finally, objects were identified by the semantic category they belong to^11^.

First, we assessed the collinearity between the three models, expressed as the Spearman correlation between the model RDMs (Fig. 2B). The low-level and categorical models have a correlation of ρ = 0.16, the shape model has ρ = 0.09 correlation with the categorical model, and ρ = 0.08 correlation with the low-level one.

**Figure 3:**
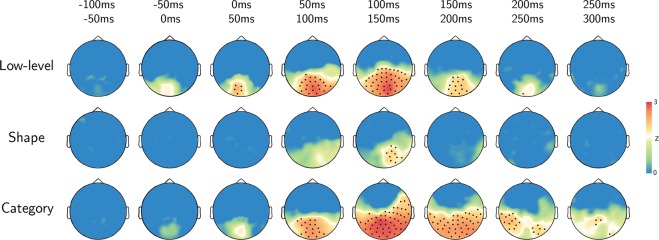
Results. Topographic plots of the group-level z-maps. Top-row reports the time bin. Black dots stand for significant channels within all the time-bin (p < 0.05, rank test, 100,000 permutations, TFCE corrected).

Then, RWA was performed within a sensor space searchlight, resulting for each subject in three maps that report the time courses of the metric ε for each sensor, i.e., the proportional contribution of each model across time. RWA controls for model multicollinearity in multiple regression: its metric (ε) does not identify the impact of each model to the prediction of a dependent variable in isolation (i.e., beta weight), as in common multiple linear regressions, but considers also how each model relates to (i.e., is correlated with) the others. Thus, it reflects in a suitable manner the proportional impact of each variable on the prediction of brain activity (Fig. 2C). The single-subject maps were aggregated in group-level z-maps for each model, corrected for multiple comparisons and divided in 50 ms-long time bins for displaying purposes. Only the sensors whose corrected z-values were significant in the entire bin were retained, as displayed in Fig. 3 (black dots mark significant sensors: p < 0.05, rank test, 100,000 permutations, TFCE corrected).

Results show that the model based on low-level features (contrast and spatial frequencies) is significant at early stages after stimulus presentation (0–50 ms) in a cluster of posterior and medial sensors. This cluster expands in the lateral and anterior directions, reaching a maximum in the 100–150 ms interval, when most of the posterior sensors are significant. Shape features are instead restricted to a right posterior location in the 100–150 ms interval, and do not reach significance in the remainder of sensors and time bins. The category-based model is significant in medial and posterior sensors starting at 50–100 ms. The cluster expands to most of the posterior and lateral sensors, with a maximum spatial extent between 100 and 200 ms, then restricting to the posterior and lateral sensors in the 200–250 ms time bin. A cluster of right posterior sensors shows significant weights for the three models in the 100–150 ms time bin only. None of the models was significant in the remaining parts of the time course (before stimulus onset and after 300 ms).

Even if the task was intended to orient subjects’ efforts specifically towards high-level semantic processing, attention towards local features could account for the observed results. To this aim, we compared the responses between semantically similar and dissimilar stimuli and found no significant difference (p > 0.20; see Supplementary Fig. S1). Thus, results are likely not driven by task demand.

## Discussion

The visual machinery is a general-purpose system, relying on different representations that often are collinear or interact to each other. Here, by taking into account model collinearity, we revealed the spatiotemporal dynamics of joint feature processing within the human visual system, to assess the relative contribution of low-level, shape and category features in predicting MEG-based representations. We observed both a temporal and spatial co-occurrence of low-level, shape and categorical processing, early in time (100–150 ms) in posterior sensors. Specifically, we showed that (a) low-level features (i.e., contrast and spatial frequencies) are processed early (0–50 ms) after stimulus onset within posterior MEG sensors, spreading in time from medial to lateral locations; (b) shape coding is limited within a few right posterior sensors in a brief time window (100–150 ms) and co-occurs with low-level and categorical processing; (c) categorical representation emerges later than the onset of low-level processing and is more prolonged, but spreads within a similar pattern of sensors.

Our results demonstrate that within 100–150 ms after stimulus onset, these features are processed concurrently, suggesting that object discrimination may result from independent parallel processing (i.e., orthogonal feature-based descriptions processed with similar temporal dynamics), rather than from a strict feed-forward hierarchy. The observed spatiotemporal overlap is in line with previous neuroimaging evidence showing that category and shape are processed within the same visual regions^15,21^, and can be decoded in the 130–200 ms time window within the high-level visual cortex, as shown in a combined fMRI-MEG study, which focused on body parts and clothes^22^. Here we employed a model-based approach which also embedded low-level features, and sampled stimuli from a broader set of categorical classes. In addition, we introduced RWA to overcome multicollinearity, which was not explicitly addressed in previous studies.

Of note, our results raise questions concerning the role of shape in categorization. The synchronization between the three models in our data occurs in a time window (100–150 ms) that overlaps with those of perceptual organization (70–130 ms) and categorical recognition of visual information (>130 ms), as indicated by previous neurophysiological and functional studies in both human and nonhuman primates^23–28^.

Whether shape processing is needed to recognize and classify objects in a scene has not been clarified yet. The classical view that considered shape essential to recognition^29^ has, however, being challenged by the success of several appearance-based computational models that could perform object recognition by relying on low-level features only^30^. Since object segmentation occurs during passive natural image viewing^31^ and controls scene reconstruction^25^, shape analysis can be similarly triggered by object viewing also in a task for which shape is not explicitly relevant. Thus, our observation has at least two possible explanations: (a) shape processing is to some extent necessary for categorization or, alternatively, (b) it is not, but it is an automatic process occurring even when not overtly required by the task. The former hypothesis may, however, not be consistent with our results that show categorical representations occurring earlier than shape-based representations. In addition, the latter case would be in line with evidence suggesting that the extraction of object affordances – i.e., shape-related features which are able to facilitate or even trigger actions – is a fast and automatic process^32,33^. However, a conclusion on this topic can be reached only by further studies involving task modulation^34^. Of note, task is able to influence the strength of object processing late in time (>150 ms^35^:).

Another interesting result is the early emergence (50–100 ms) of categorical processing within the same pattern of sensors that also encode contrast and spatial frequencies. As mentioned before, object recognition has been described as occurring at 150 ms or later^28^. We observed category representations within posterior sensors well before (even accounting for the temporal smoothing potentially introduced by the searchlight procedure). Early occurrence of categorical processing has been observed also in previous MEG studies^16,35^.

In the past years, mounting evidence revealed a top-down control of neurons in the early visual cortex^24,36–40^. Moreover, in a series of elegant studies^25,41,42^, Neri found psychophysical evidence of a top-down predictive mechanism, comprising a progressive refinement of local image reconstruction driven by global saliency or semantic content. At the macroscale, the effects of this mechanism imply that both local (i.e., low-level features) and global (i.e., object-related) representations should be retrieved early in time (<150 ms) within the visual cortex. Our results, show early (from 50 to 200 ms), overlapping patterns for low-level and categorical processing in posterior MEG sensors, in line with this view. However, further research is needed to directly test the causal role of top-down feedbacks in controlling low-level processing within the occipital cortex, which falls beyond the original scope of this work.

A further general remark should be made. As mentioned before, multicollinearity is a pervasive property of our surrounding environment. Indeed, one of the most fascinating features of our visual system is the way it deals with correlated statistics within the natural domain, to optimally represent the retinal input^43^, and to make sense of the external world, through the mean of learning and generalization. Indeed, visual correspondences are the mechanism we used to evolve more abstract, categorical representations^44^. However, from the researcher perspective, this leads to an extreme effort in balancing dimensions of interest, or in developing orthogonal models. In addition, two further aspects should be considered: first, as shown empirically^13^, since different stimuli typically vary within multiple dimensions simultaneously, it is almost impossible to isolate a single dimension of interest; second, the effort in building orthogonal competing descriptions increases with the number of tested models.

Several methods have been proposed to overcome models collinearity (for a review, see^45^:). Within the field of neuroimaging, Lescroart, *et al*.^46^ employed a variance partitioning approach (the same method, in the domain of multiple linear regression, is known as commonality analysis – as also employed in the MEG field^35^), which aims at determining the explained variance for any possible subset of the models. While this analysis is able to estimate the variance unique to each partition, its main drawback is that partitions grow exponentially with the number of models: since there are ${2}^{p}-1$ subsets for *p* predictors, just exploring the impact of 5 models generates 31 different subsets. In light of this, even comparing a low number of models would end up in a computationally intensive process and in the challenging task of interpreting and discussing a huge number of sub-models. Moreover, the partitions related to variance shared by different models can occasionally be negative, and the interpretation of these negative components is still matter of debate^47^. From this perspective, RWA is an attractive alternative, as it estimates the relative, non-negative weight of each model and does not imply to discuss more models or components than those initially considered.

Indeed, relative weights reflect in a suitable manner the proportional impact of each variable on the prediction of brain activity and - if the predictors are standardized - sum up to the total explained variance^17^. However, some limitations also affect RWA: the most relevant is that estimated weights are not invariant to the orthogonalization procedure employed. Though, it has been proven that, the more the orthogonal variables approximate the original variables, the more reliable the estimated weights become (for a deeper treatment of the topic, see^17^:). Therefore, RWA may represent a fast and appealing recipe to deal with model multicollinearity within the neuroimaging field, especially when three or more models are compared.

In conclusion, this study reveals the spatiotemporal dynamics of object processing from a model-based perspective, providing evidence in favour of an integrated perceptual mechanism in object representation.

## Methods

### Participants

Sixteen healthy right-handed volunteers (5F, age 27 ± 2) with normal or corrected to normal visual acuity participated in the study. All subjects gave informed consent to the experimental procedures and received a monetary reward for their participation. The study was approved by the Ethics Committee for research involving human participants at the University of Trento, and all the experimental procedures were conducted in accordance with the Declaration of Helsinki.

### Stimuli

Visual stimuli were colour pictures representing thirty different objects from six semantic categories (fruits, vegetables, animals, birds, tools, vehicles). The set of stimuli were used in two previous fMRI studies from our group^9,12^, and were controlled for psycholinguistic features and familiarity (for details, see^12^). Stimuli were presented using MATLAB and the Psychophysics Toolbox^48^, and were projected on a translucent screen placed at about 130 cm from the participant, using a Propixx DLP projector (VPixx technologies), with a refresh rate of 60 Hz and a resolution of 1280 × 1024 pixels (21.7 × 13.16°).

### Task and design

The experiment was organized in eight runs, each consisting of three blocks (see Fig. 2A). In each block, the thirty images were presented in randomized order, and participants were engaged in a semantic judgment task to ensure that they focused the attention on the stimuli^49^. At the beginning of each block, a binary target question (e.g., “Is it a tool?”) was shown; once subjects read the questions, they prompted the start of the block by pressing a button on a keyboard. Within each block, subjects answered (yes/no) to the question presented at the beginning using the keyboard. All pictures were presented 24 times, with a different target question for each repetition. 5 s-long resting periods preceded and followed each block, and 1 s-long resting periods followed the behavioural response to each stimulus within a block. During the resting periods, subjects had to fixate a black cross, displayed in the centre of the screen. The order of the questions was randomized across participants.

### Models

In order to predict MEG representational geometries, three different descriptions were built, representing different physiologically relevant properties of the objects seen by the subjects (see Fig. 2B). First, a low-level model, which captures the arrangement of spatial frequencies in a V1-like fashion, was employed: a GIST^30^ descriptor for each stimulus was derived by sampling (in a 4 × 4 grid) the responses to a bank of isotropic Gabor filters (8 orientations and 4 scales). The descriptor (consisting of a vector with 512 elements) of each stimulus was then normalized and compared to each other stimulus using the pairwise correlation distance (1 – Pearson’s r). Second, a shape model was computed. Similarly to previous neuroimaging investigations on the same topic^9,20^, the medial-axis transform^19^ was extracted from each manually segmented and binarised object silhouette. Then, shock-graphs skeletal representations were built, and their pairwise dissimilarity was computed using the ShapeMatcher algorithm (http://www.cs.toronto.edu/~dmac/ShapeMatcher/; Van Eede, *et al*.^50^), which estimates the minimum deformation needed in order to match two different shapes^51^. Finally, the thirty stimuli were described based on their semantic category, obtaining a binary categorical model.

### MEG data acquisition

MEG data were recorded using an Elekta VectorView system with 306-channels, 204 first order planar gradiometers and 102 magnetometers (Elekta-Neuromag Ltd., Helsinki, Finland), located in a magnetically shielded room (AK3B, Vakuumschmelze, Hanau, Germany). The sampling rate was 1 kHz. Head shapes were recorded from each participant immediately before the experiment, using a Polhemus Fastrak digitizer (Polhemus, Vermont, USA) recording the position of fiducial points (nasion, pre-auricular points) and around 500 additional points on the scalp. MEG data were synchronized with experiments timing by sending four different triggers at question presentation, first button press (after question), stimulus presentation and stimulus-related behavioural responses (button presses), respectively.

### MEG data pre-processing

MEG data pre-processing was performed using the Fieldtrip toolbox^52^. First, a bandpass (1–80 Hz) and a notch (50 Hz) 4^th^ order Butterworth IIR filters were applied to the data^53^. Filtered signals were then cut in epochs from 500 ms before to 1 s after stimulus onset and resampled at 400 Hz. Subsequently, data were visually inspected according to a set of summary statistics (range, variance, maximum absolute amplitude, maximum z-value) to search for trials and channels affected by artefacts, using the procedure for visual artefact identification implemented in Fieldtrip; trials marked as bad were rejected and noisy sensors were reconstructed by interpolating their spatial neighbours. On average, 8% of the trials and 10% of the channels were rejected for each subject.

### Searchlight analysis

A searchlight analysis was performed using CoSMoMVPA^54^, retaining the MEG data from the gradiometers only. First, the time-locked patterns for the individual trials were reduced to thirty pseudo-trials (one for each stimulus)^55^. Searchlights were then defined for each time point of the pseudo-trials using a spatial and temporal neighbouring structure^56^. Each searchlight included 10 dipoles (pairs of combined gradiometers) in the spatial domain, and each time point plus the ten preceding and following it (i.e., 21 time points, 52.5 ms) in the temporal domain. Within each spatiotemporal searchlight, a time-varying representational dissimilarity matrix (RDM) was derived for the MEG data by computing the pairwise correlation distances between pattern of responses to the thirty stimuli^57^; prior to computing the RDM, stimulus-specific activity patterns were normalized (z-scored).

### Relative weights analysis (RWA)

In order to estimate how well each model RDM was related to MEG representational geometries, a multiple linear regression for each subject and each spatiotemporal searchlight was performed. Since some of the three models RDMs are significantly correlated the Relative Weights Analysis (RWA), introduced by Johnson^17^, was adopted. The metric on which RWA relies is called epsilon (ε) and reflects both the unique contribution of each model and its impact when all the other models are considered.

The RWA procedure is graphically synthetized in Fig. 2C. Basically, the models RDMs were first orthogonalized, by performing a Principal Component Analysis (PCA), and the RDMs from each spatiotemporal searchlight were regressed on the so obtained orthogonal versions of the models RDMs. Then, the regression coefficients were related back to the original model RDMs by regressing the orthogonal RDMs also on the models RDMs. Finally, for the *j-th* model, epsilon was calculated as:

$${\varepsilon }_{j}=\sum _{k=1}^{p}{\lambda }_{jk}^{2}{\beta }_{k}^{2}$$

where *p* is the number of models, ${\beta }_{k}^{2}$ is the variance (i.e., the squared standardized regression coefficient) in each searchlight RDM accounted for by the *k-th* orthogonal RDM, and ${\lambda }_{jk}^{2}$ is the variance in the *j-th* model accounted for by the *k-th* orthogonal RDM.

### Statistical analyses

The RWA analysis, performed within the spatiotemporal searchlights as described above, provided a time course of the metric (ε) for each sensor and time point. To estimate the group-level spatiotemporal distribution of weights for each of the three models, a one sample non-parametric test was performed, using a null distribution generated with 100,000 permutations (rank test), as implemented in CoSMoMVPA. Correction for multiple comparisons was made at cluster-level using a threshold-free method (TFCE^58,59^:). Z-values corresponding to a corrected p-value of 0.05 (one-tailed) were considered significant.

## Supplementary information

Supplementary Material

## Data Availability

The datasets generated during and/or analysed during the current study are available from the corresponding author on reasonable request.

## References

[1] Malcolm GL, Groen IIA, Baker CI (2016). Making Sense of Real-World Scenes. Trends Cogn Sci.

[2] Olshausen BA, Field DJ (1996). Emergence of simple-cell receptive field properties by learning a sparse code for natural images. Nature.

[3] Vinje WE, Gallant JL (2000). Sparse coding and decorrelation in primary visual cortex during natural vision. Science.

[4] Rice GE, Watson DM, Hartley T, Andrews TJ (2014). Low-level image properties of visual objects predict patterns of neural response across category-selective regions of the ventral visual pathway. J Neurosci.

[5] Groen, II *et al*. Distinct contributions of functional and deep neural network features to representational similarity of scenes in human brain and behavior. *Elife* **7**, 10.7554/eLife.32962 (2018). 10.7554/eLife.32962 PMC5860866 29513219

[6] Lescroart MD, Biederman I (2013). Cortical representation of medial axis structure. Cereb Cortex.

[7] Carlson ET, Rasquinha RJ, Zhang K, Connor CE (2011). A sparse object coding scheme in area V4. Curr Biol.

[8] Hung CC, Carlson ET, Connor CE (2012). Medial axis shape coding in macaque inferotemporal cortex. Neuron.

[9] Handjaras G (2017). Modality-independent encoding of individual concepts in the left parietal cortex. Neuropsychologia.

[10] Haxby JV (2001). Distributed and overlapping representations of faces and objects in ventral temporal cortex. Science.

[11] Kriegeskorte N (2008). Matching categorical object representations in inferior temporal cortex of man and monkey. Neuron.

[12] Handjaras G (2016). How concepts are encoded in the human brain: A modality independent, category-based cortical organization of semantic knowledge. NeuroImage.

[13] Kay, K. N. Understanding visual representation by developing receptive-field models. *Visual population codes: Towards a common multivariate framework for cell recording and functional imaging*, 133–162 (2011).

[14] Torralba A, Oliva A (2003). Statistics of natural image categories. Network: computation in neural systems.

[15] Bracci S, Op de Beeck H (2016). Dissociations and Associations between Shape and Category Representations in the Two Visual Pathways. J Neurosci.

[16] Clarke A, Devereux BJ, Randall B, Tyler LK (2015). Predicting the Time Course of Individual Objects with MEG. Cereb Cortex.

[17] Johnson JW (2000). A Heuristic Method for Estimating the Relative Weight of Predictor Variables in Multiple Regression. Multivariate Behav Res.

[18] Kubilius J, Wagemans J, Op de Beeck HP (2014). A conceptual framework of computations in mid-level vision. Front Comput Neurosci.

[19] Blum H (1973). Biological shape and visual science. I. J Theor Biol.

[20] Leeds DD, Seibert DA, Pyles JA, Tarr MJ (2013). Comparing visual representations across human fMRI and computational vision. J Vis.

[21] Proklova D, Kaiser D, Peelen MV (2016). Disentangling Representations of Object Shape and Object Category in Human Visual Cortex: The Animate-Inanimate Distinction. J Cogn Neurosci.

[22] Kaiser D, Azzalini DC, Peelen MV (2016). Shape-independent object category responses revealed by MEG and fMRI decoding. J Neurophysiol.

[23] Poort J, Self MW, van Vugt B, Malkki H, Roelfsema PR (2016). Texture Segregation Causes Early Figure Enhancement and Later Ground Suppression in Areas V1 and V4 of Visual Cortex. Cereb Cortex.

[24] Williford Jonathan R., von der Heydt Rüdiger (2016). Figure-Ground Organization in Visual Cortex for Natural Scenes. eneuro.

[25] Neri P (2017). Object segmentation controls image reconstruction from natural scenes. PLoS Biol.

[26] DiCarlo JJ, Zoccolan D, Rust NC (2012). How does the brain solve visual object recognition?. Neuron.

[27] Johnson JS, Olshausen BA (2003). Timecourse of neural signatures of object recognition. J Vis.

[28] Bar M (2003). A cortical mechanism for triggering top-down facilitation in visual object recognition. J Cogn Neurosci.

[29] Biederman I (1987). Recognition-by-Components - a Theory of Human Image Understanding. Psychological Review.

[30] Oliva A, Torralba A (2001). Modeling the shape of the scene: A holistic representation of the spatial envelope. International journal of computer vision.

[31] Papale Paolo, Leo Andrea, Cecchetti Luca, Handjaras Giacomo, Kay Kendrick N., Pietrini Pietro, Ricciardi Emiliano (2018). Foreground-Background Segmentation Revealed during Natural Image Viewing. eneuro.

[32] Craighero L, Fadiga L, Umilta CA, Rizzolatti G (1996). Evidence for visuomotor priming effect. Neuroreport.

[33] Grezes J, Tucker M, Armony J, Ellis R, Passingham RE (2003). Objects automatically potentiate action: an fMRI study of implicit processing. Eur J Neurosci.

[34] Harel A, Kravitz DJ, Baker CI (2014). Task context impacts visual object processing differentially across the cortex. Proc Natl Acad Sci USA.

[35] Hebart MN, Bankson BB, Harel A, Baker CI, Cichy RM (2018). The representational dynamics of task and object processing in humans. eLife.

[36] Lamme VA, Roelfsema PR (2000). The distinct modes of vision offered by feedforward and recurrent processing. Trends Neurosci.

[37] Lamme VA (1995). The neurophysiology of figure-ground segregation in primary visual cortex. J Neurosci.

[38] Poort J (2012). The role of attention in figure-ground segregation in areas V1 and V4 of the visual cortex. Neuron.

[39] Qiu FT, Sugihara T, von der Heydt R (2007). Figure-ground mechanisms provide structure for selective attention. Nat Neurosci.

[40] Hesse JK, Tsao DY (2016). Consistency of Border-Ownership Cells across Artificial Stimuli, Natural Stimuli, and Stimuli with Ambiguous Contours. J Neurosci.

[41] Neri P (2014). Semantic control of feature extraction from natural scenes. J Neurosci.

[42] Neri P (2011). Global properties of natural scenes shape local properties of human edge detectors. Front Psychol.

[43] Olshausen BA, Field DJ (1996). Natural image statistics and efficient coding. Network.

[44] Tenenbaum JB, Kemp C, Griffiths TL, Goodman ND (2011). How to Grow a Mind: Statistics, Structure, and Abstraction. Science.

[45] Nimon KF, Oswald FL (2013). Understanding the results of multiple linear regression: Beyond standardized regression coefficients. Organizational Research Methods.

[46] Lescroart MD, Stansbury DE, Gallant JL (2015). Fourier power, subjective distance, and object categories all provide plausible models of BOLD responses in scene-selective visual areas. Front Comput Neurosci.

[47] Ray‐Mukherjee J (2014). Using commonality analysis in multiple regressions: a tool to decompose regression effects in the face of multicollinearity. Methods in Ecology and Evolution.

[48] Brainard DH (1997). The Psychophysics Toolbox. Spat Vis.

[49] Sudre G (2012). Tracking neural coding of perceptual and semantic features of concrete nouns. Neuroimage.

[50] Van Eede, M., Macrini, D., Telea, A., Sminchisescu, C. & Dickinson, S. S. 64–69 (IEEE).

[51] Sebastian TB, Klein PN, Kimia BB (2004). Recognition of shapes by editing their shock graphs. IEEE Trans Pattern Anal Mach Intell.

[52] Oostenveld R, Fries P, Maris E, Schoffelen JM (2011). FieldTrip: Open source software for advanced analysis of MEG, EEG, and invasive electrophysiological data. Comput Intell Neurosci.

[53] Gross J (2013). Good practice for conducting and reporting MEG research. Neuroimage.

[54] Oosterhof NN, Connolly AC, Haxby JV (2016). CoSMoMVPA: Multi-Modal Multivariate Pattern Analysis of Neuroimaging Data in Matlab/GNU Octave. Front Neuroinform.

[55] Guggenmos M, Sterzer P, Cichy RM (2018). Multivariate pattern analysis for MEG: A comparison of dissimilarity measures. Neuroimage.

[56] Su, L., Fonteneau, E., Marslen-Wilson, W. & Kriegeskorte, N. Spatiotemporal searchlight representational similarity analysis in EMEG source space. In *Second International Workshop on Pattern Recognition in NeuroImaging*. IEEE, 97–100 (201).

[57] Kocagoncu E, Clarke A, Devereux BJ, Tyler LK (2017). Decoding the Cortical Dynamics of Sound-Meaning Mapping. J Neurosci.

[58] Smith SM, Nichols TE (2009). Threshold-free cluster enhancement: addressing problems of smoothing, threshold dependence and localisation in cluster inference. Neuroimage.

[59] Pernet CR, Latinus M, Nichols TE, Rousselet GA (2015). Cluster-based computational methods for mass univariate analyses of event-related brain potentials/fields: A simulation study. J Neurosci Methods.

