# Supplementary material for: Common spatiotemporal processing of visual features shapes object representation
Source: Sci Rep. 2019 May 20;9:7601. doi: 10.1038/s41598-019-43956-3 (PMC6527710; doi:10.1038/s41598-019-43956-3)
Supplement: Supplementary file 1 — Supplementary Material [file 41598_2019_43956_MOESM1_ESM.docx]

**Supplementary Information for:**

**Common spatiotemporal processing of visual features shapes object representation**

Paolo Papale^1^, Monica Betta^1^, Giacomo Handjaras^1^, Giulia Malfatti^2^, Luca Cecchetti^1^, Alessandra Rampinini^1^, Pietro Pietrini^1^, Emiliano Ricciardi^1^, Luca Turella^2^ and Andrea Leo^1*^

^1^ Momilab, IMT School for Advanced Studies Lucca, 55100, Lucca, Italy

^2^ Center for Mind/Brain Sciences (CIMeC), University of Trento, 38068, Trento, Italy

* Corresponding author: [*andrea.leo@imtlucca.it*](mailto:pietro.pietrini@imtlucca.it)


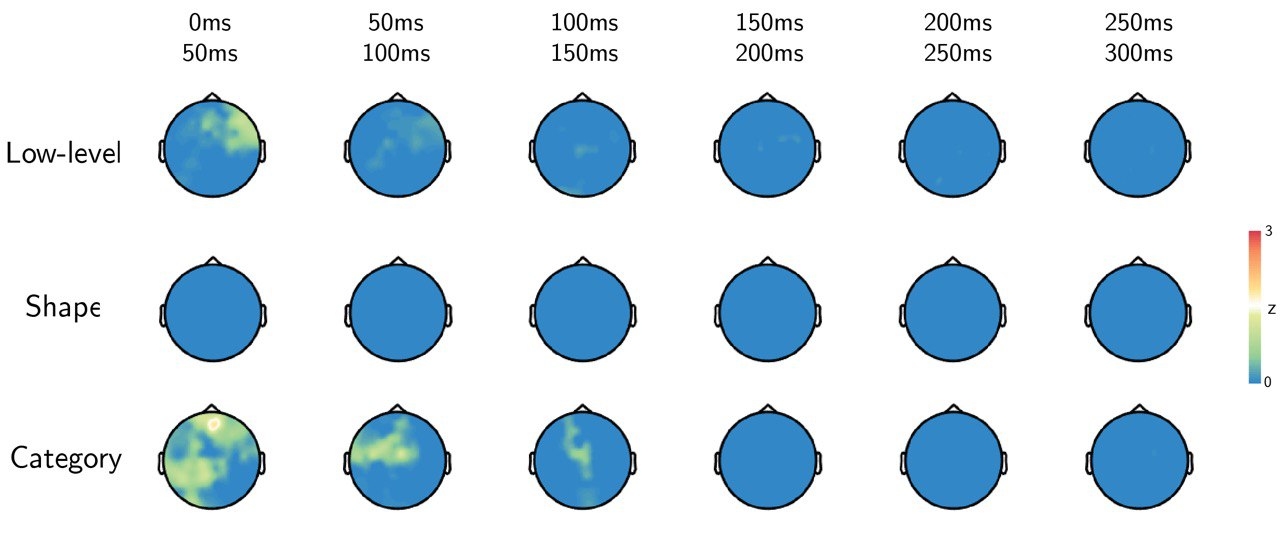


**Supplementary Figure S1**

Differences between the representation of stimulus features in MEG activity for visually and semantically similar as compared to dissimilar items. The maps report the sensor patterns and time bins in which the weights for each of the three models were higher for similar than dissimilar items (paired rank test, p<0.2). Our experimental paradigm aimed to reveal *whether* and *when* low-level, global shape or categorical information are independently processed, while participants were engaged in a semantic judgment task that does not require explicit attention to object shape. However, even if the task was intended to orient subjects’ efforts specifically towards high-level semantic processing, it is important to rule out the potential bias on participants’ attention towards local features. To this purpose, we compared the responses between semantically similar and dissimilar stimuli. We partitioned the MEG representation dissimilarity matrices (RDMs) as pertaining to visually and semantically similar (e.g., *fruits vs. vegetables, animals vs. birds, tools vs. vehicles)* or dissimilar (e.g., *vegetables vs. animals, birds vs. fruits, animals vs. vehicles*) comparisons. Since the number of dissimilar comparisons is greater than the number of similar ones, we randomly selected three dissimilar comparisons from the RDMs, to balance the similar ones. Then, we replicated the RWA and the identification of group-level spatiotemporal clusters, as described in the main text, on these partitions of RDMs, and performed a paired rank test between similar and dissimilar conditions for each model. As evident in the topographic plots, there are no significant differences between similar and dissimilar comparisons in any combination of sensors and time-bins. For this reason, we conclude that our results are likely not driven by the perceptual or semantic (dis)similarity between stimuli, excluding therefore a role of task demand.
